# The Effects of a Multidimensional Exercise Program on Health Behavior and Biopsychological Factors in Mexican Older Adults

**DOI:** 10.3389/fpsyg.2019.02668

**Published:** 2020-01-24

**Authors:** Norma Angélica Borbón-Castro, Andrés Aquilino Castro-Zamora, Rosa María Cruz-Castruita, Nancy Cristina Banda-Sauceda, Manuel Francisco De La Cruz-Ortega

**Affiliations:** ^1^Programa Educativo Licenciado en Entrenamiento Deportivo, Universidad Estatal de Sonora, Hermosillo, México; ^2^Programa Educativo Licenciado en Nutrición Humana, Universidad Estatal de Sonora, Hermosillo, México; ^3^Facultad de Organización Deportiva, Universidad Autónoma de Nuevo León, San Nicolás de los Garza, México; ^4^Facultad de Nutrición y Salud Pública, Universidad Autónoma de Nuevo León, San Nicolás de los Garza, México

**Keywords:** older adults, multidimensional exercise programs, healthy aging, physical and psychological well-being, depression

## Abstract

**Background:** The population of older adults is increasing worldwide, which brings attention to the importance of healthy aging. Adoption of healthy lifestyle activities such as participating in physical activity on a daily basis is key to maintaining physical and mental health. The aim of this study was to investigate the effects of participation in a 12-week multidimensional exercise program on health behavior and biopsychological factors of older adults living in Northeastern Mexico.

**Methods:** A quasi-experimental study was conducted with 45 older adults (35 females and 10 males; *M* = 67.24 ± 5.73 years). The participants were assigned to an experimental group (EG; *n* = 23) that participated in a 12-week exercise program and a control group (CG; *n* = 22). Pre- and post-analyses of the exercise intervention data were carried out to investigate the participants’ health-related variables including physical activity levels, blood pressure, self-esteem, depressive symptoms, and blood lipids profiles.

**Results:** The results indicated that the exercise intervention contributed to significant improvements in the older adults’ health-related variables for the EG when contrasted with the control group. For instance, the EG significantly improved systolic (*p* < 0.001) and diastolic (*p* < 0.027) blood pressure, blood lipids [e.g., cholesterol (*p* < 0.05)], triglycerides (*p* < 0.05), self-esteem (*p* < 0.005), and depressive symptoms (*p* < 0.002) as well as physical activity (*p* < 0.001) levels. The results also demonstrated that only those individuals in the EG diagnosed with disease benefited from improved self-esteem and physical activity levels when contrasted with their healthy counterparts.

## Introduction

As the global population of older adults continues to increase, activities such as participation in physical activity have been found to be essential to promote healthy aging and quality of life. In fact, a lack of physical activity may result in severe health and social problems (World Health Organization, 2010). Health problems may include suffering from chronic diseases (Dunlop et al., 2015; Mama et al., 2015) or depression, which can increase morbidity and mortality (Voinov et al., 2013). Social problems may include concerns about raising social and economic costs due to increases in medical expenses for older adults with health problems (Unsar and Sut, 2010) and/or development of negative stereotypes toward the older population in society (Lamont et al., 2015). These health and social impacts may result in older populations’ negative self-perception and low self-esteem that may cause loss of function and decreases in the quality of life (Unsar and Sut, 2010).

Although physical activity participation has been found to be a critical option to achieve healthy aging, the level of physical activity among older adults, in general, is low (McPhee et al., 2016). In Mexico, only 12.2% of older adults engage in a recommended amount of physical activity (Ramos, 2016). Such a low level of physical activity can be correlated easily with physical and psychological health issues for this age group. Specifically, a lack of physical activity tends to increase risk of cardiovascular diseases, cancer, and metabolic syndrome (Murrock and Graor, 2014; Pérez, 2014; McPhee et al., 2016). For psychological issues, approximately 30% of older adults who live independently suffer from depression (Murrock and Graor, 2014). It should be noted that physical and psychological health are interrelated and affect one another. As one example, being diagnosed with depression can be associated with decline in physical health such as chronic diseases (Bordon, 2012), loss of function (Unsar and Sut, 2010), and disability (Wight et al., 2011).

Since physical and psychological well-being play a significant role in maintaining and improving older adults’ health, integrating multidimensional factors into a health promotion program offers a clear path to improving and protecting the health and general well-being of older adults. In particular, including holistic approaches in an exercise program can improve opportunities for older adults to initiate and continue exercise compared with programs that do not consider biological (age, sex, and others); social (such as ethnicity, and socioeconomic status); psychological well-being, social support, motivational readiness, self-efficacy, and others factors (Pender et al., 2011; Mama et al., 2015, 2017). Research has found that it is important that an exercise program places emphasis on satisfying interpersonal, organizational, and environmental factors (Glanz and Bishop, 2010; De Silva et al., 2014) such as promoting enjoyment and interaction among exercise participants while increasing accessibility and safety at exercise facilities (Sowle et al., 2017).

Integrating multidimensional factors (e.g., biopsychosocial factors) into health promotion programs helps participants successfully enhance health-related behavior (e.g., participating in exercise; Pender et al., 2011; Mama et al., 2017). Theories and models focusing on such integrative approaches have been established and utilized to design, implement, and evaluate health promotion programs (Lippke and Ziegelmann, 2008; Bartholomew et al., 2011). As one example, the Health Promotion Model (HPM; Pender et al., 2011) claims that each individual tries to regulate his or her own behavior based on his or her characteristics, experiences, and behavior-specific knowledge and affect. Health-promoting behavior – which is considered the desired behavioral outcome and the end point in this model – should contribute to obtaining health benefits such as improved health, enhanced functional ability, and better quality of life. The HPM considers compliance with multidimensional factors such as age, gender, physical abilities, perceived competence or self-efficacy are crucial for people who register a change in behavior that promotes health, such as participating in the exercise activities.

In another model established by Bouchard et al. (1994), physical activity levels play a significant role in health-related fitness (e.g., cardiorespiratory level, muscular strength) and the overall health status. Other variables that would affect these factors include the individuals’ heredity and lifestyle (e.g., diet patterns, smoking) and physical and social environments. For example, sociocultural and economic factors could influence an individuals’ participation in physical activity, thus affecting their fitness and health status. As noted previously, there is strong evidence that regular participation in physical activity is beneficial for physical and psychological health, including factors such as improvements in hypertension, type 2 diabetes mellitus, depression, and anxiety (Shephard, 1995; Carek et al., 2011; Gil et al., 2017; Alomoto et al., 2018; Solórzano and Vargas, 2019).

Sometimes the programs that reach citizens are far from having the required planning and structuring, or they are not supported by clear scientific support. In relation to this point, the differences and benefits between the “Structured-Unstructured” programs are that the former can be based and evaluated from theories or theoretical models that emphasize the adequacy of facilitating environments to enhance the adherence of the people to an active lifestyle and explain the processes that mediate or condition the causal relationship between the program and the desired results (Frieden, 2010; Glanz and Bishop, 2010; Bartholomew et al., 2011). Although the aging of the population occurs worldwide, the difference between countries lies in the planning and preparation to face this change. It is here that the implementation of specific physical activity programs for older adults becomes essential to improve current health conditions (Riekert et al., 2013).

Based on the information previously mentioned, the aim of this study was to investigate the effects of the participation in a 12-week multidimensional exercise program on health behavior considering as the physical activity levels and the biological factors of blood profiles (glucose, glycosylated hemoglobin, cholesterol, triglycerides, high-density lipoproteins, low-density lipoproteins, and very low-density lipoproteins), body weight, body mass index, blood pressure, and nutritional status; psychological factors of depression and self-esteem; and the social factors of sociodemographic characteristics and socioeconomic level of older adults living in northeastern Mexico. We considered, for the design of the exercise program, the healthy fitness factors mentioned in the model of Bouchard et al. (1994) and social factors of HPM. To evaluate its effectiveness, the biological and psychological personal factors as the health promoting behavior of the HPM were considered (HPM: Pender et al., 2011).

## Materials and Methods

### Sample and Inclusion/Exclusion/Elimination Criteria

A convenience sampling was utilized based on accessibility to a sports and recreation center in the state of Sonora that is part of the facilities of Public Health Institution of Mexico. Seventy older adults who lived independently in the urban community were registered for geriatric care programs (e.g., crafts, embroidery, dance, and recreational activities) provided by the center. The STATS 2.0 software was used to estimate an appropriate sample size considering the following factors: 95% confidence interval, 5% error and 10% losses to follow-up.

Initial interviews were conducted with 50 older adults to examine if these individuals met the following inclusion criteria: able to walk 2.44 meters independently, receipt of permission from their primary doctors, and assessment for exclusionary risk factors. In order to check the participants’ risk factors, the research team utilized two approaches: collecting general information about the participants such as demographics (e.g., age, education level), brief history of disease, and use of medicine; and the completion of a more detailed health history and current health issues using the AHA/ACSM Health/Fitness Facility Preparticipation Screening Questionnaire. All 50 older adults met the criteria and were invited to participate in the study and received details of the study protocol. Forty-five older adults (35 females and 10 males; *M* = 67.24 ± 5.73) ended up accepting the invitation, signed a written informed consent, and passed medical evaluation conducted by medical doctors prior to the study. The participants included healthy individuals and those who were under medical supervision or needed treatment for the following health conditions: hypertension, type 2 diabetes mellitus, and obesity. Participants were excluded if they had cognitive impairment, had physical limitations such as pain or discomfort in the chest by increasing the level of physical exercise, angina pectoris, those who had a cardiac implant, suffered complications that affect the musculoskeletal system, had chronic diseases different from those indicated in the inclusion criteria or chronic diseases indicated in the inclusion criteria but without medical control. Older adults were eliminated from the study if they decided to leave the study and if they did not comply with 80% attendance at the exercise program. In relation to the criteria considered for the study, nine people were excluded because they had diseases other than those indicated in the inclusion criteria.

### Study Design

A quasi-experimental design was employed for this study where all of 45 participants were assigned to either experimental or control group based on activities they had engaged in at the center prior to participating in this study. As a result, 23 individuals who were enrolled in physical activity sessions offered by the center before the study were assigned to the experimental group (EG) and 22 individuals who were registered in embroidery, weaving, and handicraft activities offered by the institution were assigned to the control group (CG). The participants selected for both groups met the inclusion criteria and agreed to be part of the study. To control the effect of the program, as part of the informed consent, the GE agreed not to participate in other types of programs or physical activity other than the activities they already performed regularly and the GC agreed to continue performing the activities of the center in which they were registered; the data was confirmed at the beginning, after two months and at the end of the program through open questions stipulated in the sociodemographic survey.

The research considered the criteria for carrying out human research projects established by the Official Mexican Standard NOM-012-SSA3-2012 (Diario Oficial de la Federación [DOF], 2013) and was approved by the Comité Institucional de Bioética (Institutional Committee of Bioethics) of the Instituto Técnico de Sonora, México (Technical Institute of Sonora Mexico).

### Exercise Program Intervention

Prior to the implementation of the program, researchers designed the sessions corresponding to each module according to the recommendations for the prescription of physical activity in older adults with and without chronic disease of the experts (American College of Sports Medicine, 2013; World Health Organization, 2015), previous research (Jiménez, 2007; García, 2013; Keller et al., 2014; Loprinzi et al., 2015; Mama et al., 2015), and the theoretical background mentioned in the introduction (Bouchard et al., 1994; Pender et al., 2011). Subsequently, the sessions were modified according to the physical characteristics of the participants detected in the evaluation carried out with the Senior Fitness Test Battery (Rikli and Jones, 2001). In addition, some interpersonal factors of the participants were considered in the design of the program, including team activities and promoting communication between instructors.

Some sociocultural factors of the participants in the choice of a specific type of music that was popular in the region and culturally popular team activities such as cachibol are also considered. The popular music of the region was used to stimulate movement and accompany the exercise (Clark et al., 2016), in addition to using it to regulate the intensity of physiological arousal (Karageorghis and Priest, 2012), promote participation, enjoy exercise (Murrock and Higgins, 2009), and increase physical activity levels (Altenmüller and Schlaug, 2013).

The work team responsible for data collection and implementation of the exercise program is described below: undergraduate and master’s students in physical activity and sport, who are responsible for collecting health behavior data, imparting the sessions, and monitoring participants during them through the observation and Borg rating of perceived effort (RPE; Borg, 1982); undergraduate students in nutrition responsible for assessing nutritional status; a nurse in charge of the evaluation of blood pressure, the psychological and social factor; and a chemist in charge of taking blood tests.

The primary goal of the multidimensional exercise program was to help the participants improve aerobic capacity while aiming to assist them in improving muscular strength, speed, agility, flexibility, and coordination. Cognitive function activities also were included at least twice a week with aims to improve their memory, through group dynamics with cognitive stimulation activities. The exercise class was offered 5 days a week for 12 weeks with a total of 60 sessions. Each session lasted for 60 min, including a 10-min warm-up, a variety of exercises for 40 min, and cool-down for 10 min. The 12-weeks exercise sessions were divided in to six modules identified with letters according to the increase in the intensity of the exercise for every 2 weeks (Porter et al., 2011). In each module, a goal was established, and the exercises developed in it focused mainly on achieving it (Supplementary Table S1).

**Figure 1:**
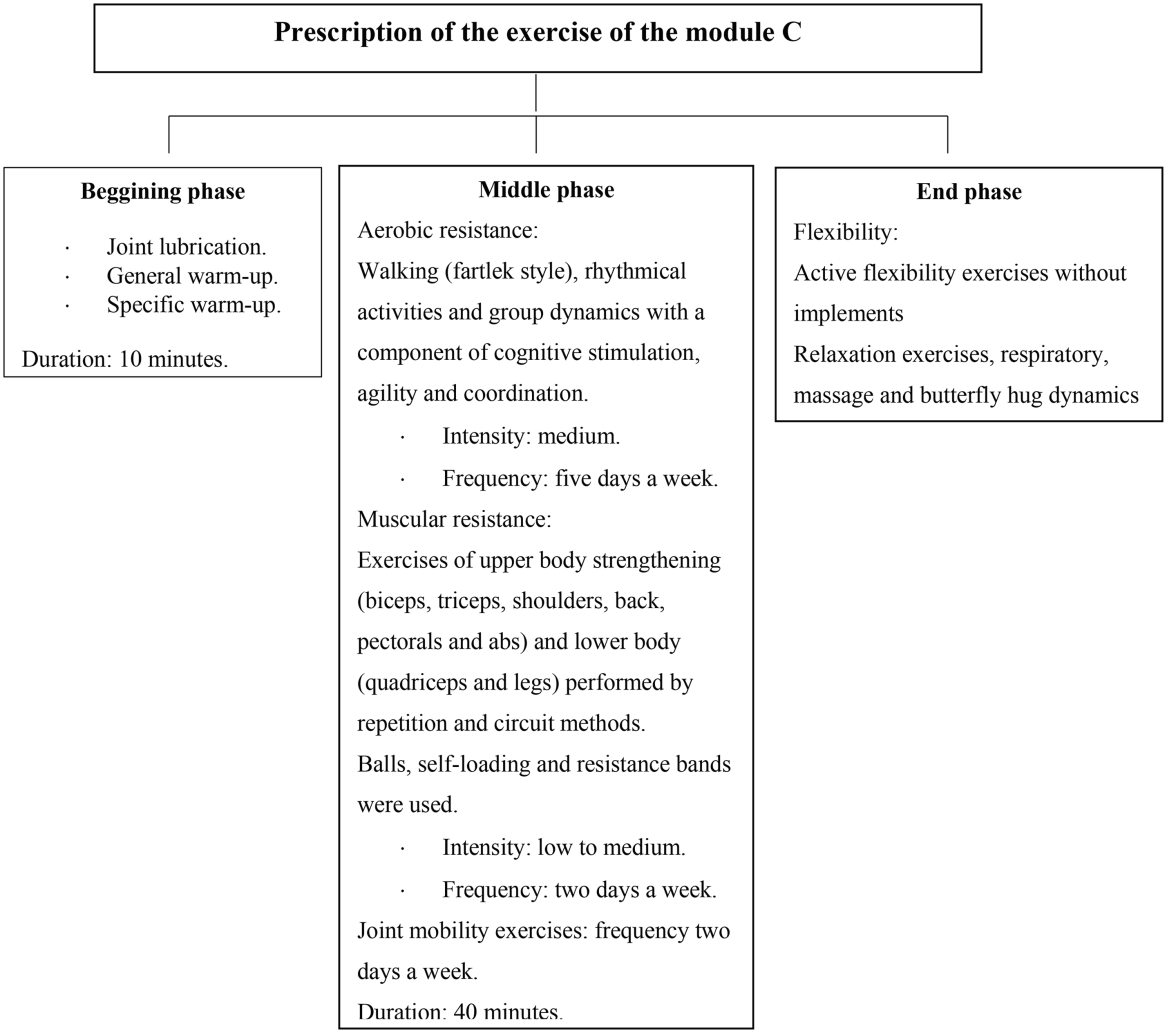
Example of prescription of exercise protocols in one of the modules of the exercise program.

During the implementation of the program, the sessions were monitored by the researchers, responsible for evaluating the development of the program through observation and a checklist of the duration of the sessions, the time of each part of the session, the intensity, the music, material and explanation of the exercises by the instructors according to schedule. The exercise protocols and intensity were changed if instructors confirmed participants’ progress through observation and/or communication. In each session, three to four exercise instructors took care of 23 participants where they took turns leading exercise instruction while other instructors observed the participants to provide feedback or make corrections whenever necessary. The instructors constantly monitored the participants’ perception of exercise intensity by RPE four times and inquiring about their physical conditions during a 60-min class. In each session, a variety of exercise modalities were used such as dancing, team activities (e.g., throwing or kicking a ball as a team), and resistance (like light walk, rhythmic activities, and ball game) and circuit training [e.g., upper train strengthening exercises (biceps, triceps, shoulder, back, pectorals, and abdomen) and lower (thigh and leg), Figure 1]. The main materials used for cognitive stimulation exercises were ribbons of different colors, scarves, hats and balloons.

### Measures

After the initial health screening was conducted, the research team carried out measurements including anthropometry, nutritional status, blood profiles, depressive symptoms, self-esteem, and physical activity levels as well as surveys for sociodemographic and socioeconomic levels. Pre- and post-intervention assessments were carried out to both groups 1 week before the beginning of the exercise program (pre) and 1 week after the conclusion (post) as follows: a blood test was provided on the first day while anthropometric psychological and socioeconomic measurements and the blood pressure and physical activit**y** assessments were conducted on the second day. The following section includes detailed descriptions of these measurements and surveys.

#### Anthropometry

A Seca 634 scale and a Seca 274 stadiometer (Seca Mexico, Mexico City) were utilized to measure body weight and height, respectively. BMI was calculated by dividing body weight by height squared (BMI = kg/m^2^).

#### Nutritional Status

The Mini Nutritional Assessment (MNA) was used to identify the nutritional status of the older adults. The MNA is composed of 18 questions in two parts: the screening and the assessment sections. The overall score resulting from the sum of the two parts reveals nutrition status (Nestle Nutrition Institute, 2016a): normal nutrition status from 24 to 30 points, risk of malnutrition from 17 to 23.5 points, and malnutrition with less than 17 points. MNA has been shown to have a sensitivity of 96%, a specificity of 98%, and a positive predictive value of 97% compared to clinical status (Nestle Nutrition Institute, 2016b).

#### Blood Profiles

A fasting blood test was conducted at the sports and recreation center to measure blood glucose levels and lipid profiles including total cholesterol, high-density lipoprotein (HDL), low-density lipoprotein (LDL), very low-density lipoprotein (VLDL), triglycerides, atherogenic index of plasma, and glycosylated hemoglobin (HbA1c). The blood sample was extracted through the Vacutainer system and later analyzed with the Analyzer of Differentiated Chemistry, Automated, Vital Scientific, Vitalab Selectra 2^®^ through the wet chemistry method.

#### Blood Pressure

A Riester nova-presameter^®^ mercury sphygmomanometer (Rudolf Riester GmbH, Jungingen, Germany) was used to measure blood pressure while following the protocol described in the Official Mexican Standard, NOM-030-SSA2-1999 (Secretaría de Salud, 2000).

#### Depressive Symptoms

The 15-item Geriatric Depression Scale (GDS-15) was used to assess if the older adults had depressive symptoms (Salguero et al., 2011; Raudsepp and Riso, 2017). This scale is composed of 15 items that require a yes or no answer and scores 0 or 1 point for each question. The total scores from 0 to 4 are considered normal, and 5 or more points are considered the presence of depression. The GDS-15 has a sensitivity of 92% and a specificity of 81% in the detection of major depression (Yesavage et al., 1982).

#### Self-Esteem

The Rosenberg Self-Esteem Scale (RSES: Rosenberg, 1965) was used to evaluate general self-esteem. This 10-item scale is considered uni-dimensional by measuring positive and negative feelings about the self, using a 4-point Likert scale that ranges from strongly agree to strongly disagree. This scale has been used to measure self-esteem in diverse populations (Zabala et al., 2016), validated in several languages including Spanish with a Cronbach’s alpha between 0.80 and 0.85 (Martín-Albo et al., 2007), and culturally adapted to the Latin context (Schmitt and Allik, 2005; Rojas-Barahona et al., 2009).

#### Physical Activity Level

The physical activity questionnaire for the older adult (CAFAM) was used to assess the participants’ physical activity level achieved at home, through participation in sport or exercise, and during free time. The questionnaire shows a reliability of 0.89 determined by the test-retest method in the older population of both genders with ages between 63 and 80 years (Voorrips et al., 1991). The participants’ physical activity level was classified using Metabolic Equivalent (METs) as follows: high >16.5 METs, moderate from 9.4 to 16.5 METs, and low <9.4 METs (Kalapotharakos et al., 2004).

#### Sociodemographic Survey

A survey was used to collect each participant’s personal data and medical history: name, sex, age, marital status, education, illnesses, medications, and affiliation to health services.

#### Socioeconomic Level

The socioeconomic level (SEL) index proposed by the Mexican Association of Marketing and Opinion Intelligence Agencies (AMAI) was used to identify the participants’ socioeconomic level by assessing their ability to satisfy their needs such as habitation, utility, technology, and education.

### Analysis

Data were analyzed using Statistica 8.0 software. Descriptive statistics were applied using central tendency and dispersion measures; the Shapiro-Wilk test was used to identify the distribution of the variables. An inferential analysis was carried out with Student’s *t*-test for independent samples and a paired *t*-test for dependent samples, only for data with normal distribution. For non-normal data distributions, nonparametric statistics were used: the Mann-Whitney *U* test for independent samples and the Wilcoxon signed-rank test for dependent samples. After the inferential analysis, an analysis was carried out to evaluate the magnitude of the change produced after the intervention with Cohen’s *d* in variables with normal distribution, considering a small effect (0.20), a medium effect (0.50), a large effect (0.80), and a very large effect (>0.80) size. The effect size analysis was complemented, with a regression analysis to see the strength of the relationship between the variables *y*.

## Results

Of the total sample (*n* = 45), 77.77% (*f* = 35) were women and 22.22% (*f* = 10) were men. Within the experimental group (EG; *n* = 23), 40% (*f* = 18) were women whereas 11.1% (*f* = 5) were men; in the control group (CG; *n* = 22), 37.7% (*f* = 17) were women, whereas 11.1% (*f* = 5) were men. The mean age was 67.78 ± 6.66 years for the EG and 66.68 ± 4.67 for the CG.

### Descriptive Statistics Regarding Biological and Social Factors

In terms of health conditions, 47.82% (*f* = 11) of the participants in the EG and 36.36% (*f* = 8) in the CG reported having two or more diseases, including high blood pressure, type 2 diabetes mellitus, and obesity. In addition, about half of the participants (EG: *f* = 13; 56.52% and CG: *f* = 8; 36.36%) reported taking two or more medications in order to control hypertension or type 2 diabetes mellitus. For social factors, there were no differences between these two groups in frequencies of using health services, types of health services used, and marital status. However, the participants in the EG tend to have higher education levels and socioeconomic status (Supplementary Table S2).

### Differences Between the Groups in the Biopsychological Factors and Health-Promoting Behavior

In terms of biological factors, only the EG showed significant improvements between pre- and post-tests in cholesterol (*p* < 0.05), triglycerides (*p* < 0.05), VLDL (*p* < 0.01), SBP (*p* = 0.001), and DBP (*p* = 0.027; Supplementary Table S3). For nutritional status, the results showed significant differences between groups (*p* < 0.01; Supplementary Table S4) such that the EG demonstrated better nutritional status compared with the CG: normal nutritional status (*f* = 19; 82.60% versus *f* = 15; 68.18%), risk of malnutrition (*f* = 04; 17.39% versus *f* = 06; 27.27%), and malnutrition (*f* = 0; 0.0% versus *f* = 1; 04.54%).

The results of the analysis of the effect size for the biological factors in the EG showed that the intervention produced a small effect in the variable of LDL (*d* = 0.48; *r* = 0.23). For the glucose (*d* = −0.46; *r* = 0.22) and nutritional status (*d* = −0.45; *r* = −0.22) variables, the effect size was small and negative, which indicates a higher than average score in the post-test. For total cholesterol (*d* = 0.63; *r* = 0.30), triglycerides (*d* = 0.70; *r* = 0.33), and VLDL (*d* = 0.78; *r* = 0.36) variables, the intervention had a medium effect on the differences found by evaluation. For diastolic blood pressure (*d* = 0.93; *r* = 0.42) effect size was large (Supplementary Table S3).

In Supplementary Table S3, the coefficient of determination confirms that in the EG for the biological factors of glucose, LDL, and nutritional status, the relationship is small (*R*^2^ = 0.048; *R*^2^ = 0.052; *R*^2^ = 0.048, respectively) for TG and VLDL the relationship it is medium (*R*^2^ = 0.108; *R*^2^ = 0.129, respectively) and for SBP (*R*^2^ = 0.409) the relationship it is large. The proportion of variance shared by the multidimensional exercise program and the SBP is 40.9% (Supplementary Table S3).

Data analysis revealed that there were statistically significant differences regarding the psychological factors. For depressive symptoms, the results showed significant differences between groups both in pre (*p* = 0.01) and post (*p* = 0.001) assessments. Depression decreased only in the EG (*p* = 0.002). For self-esteem, significant differences between the groups in the posttest were observed (*p* = 0.005); the EG demonstrated a significant increase (*p* = 0.005) between pretest and posttest (Supplementary Table S4).

In relation to the size of the difference, the scores obtained with Cohen’s *d* for self-esteem (*d* = −0.89; *r* = −0.40) is large size, which indicates that the scores obtained by the EG in the posttest are higher tha the scores obtained in the pretest. The negative value of the effect size confirms the result, that is, the average of the pretest is less than the average of the posttest. The regression analysis through the coefficient of determination for the self-esteem in the EG showed that for self-esteem the proportion of variance that is explained by the effect of the exercise program is small (*R*^2^ = 0.16; Supplementary Table S4).

The results regarding physical activity levels in METs revealed statistically significant differences between the groups at the post-intervention evaluation (*p* < 0.001), but not at the pre-intervention evaluation. Only the EG demonstrated a significant increase between pre and post assessments (*p* < 0.001). The results indicate that participating in the 12-week exercise program contributed to the participants’ engaging in more physical activities compared with those who did not participate. The scores obtained with Cohen’s *d* for the health behavior was very high and had a negative effect size (*d* = −4.24; *r* = −0.90), which indicates the average of the pretest is less than the average of the posttest. The regression analysis through the coefficient of determination for health behavior in the EG allowed to correct the negative values and showed that for the health behavior the proportion of variance that is explained by the effect of the exercise program is medium (*R*^2^ = 0.81) (Supplementary Table S4).

### Differences in Biological and Psychological Factors and Health-Promoting Behavior Between Healthy Older Adults With Disease in the Experimental Group

Supplementary Table S5 shows the results of pre and post measurements in the biological and psychological factors and health-promoting behavior between healthy older adults and those with disease within the experimental group. Both healthy older adults and those with disease improved SBP, whereas only those with disease improved self-esteem and physical activity levels.

## Discussion

The aim of this study was to evaluate the effect of the 12-week multidimensional exercise program on health behavior, the biological factors of blood profiles, body weight, body mass index, blood pressure, and nutritional status and the psychological factors of depression and self-esteem of older adults who resided in Northeastern Mexico. The results of this study showed that the older adult participants showed improved physical activity levels as well as various biopsychosocial factors such as blood lipids profiles, blood pressure, and depressive symptoms. The results are consistent with the previous research, which indicated that health promotion programs designed for the purpose of modifying behaviors allow people to adopt health behaviors (e.g., increasing physical activity levels after the exercise participation; Keller et al., 2014; Mama et al., 2015).

Regarding sociodemographic variables, the experimental group in this study tended to have higher levels of education and socioeconomic status and demonstrated higher levels of physical activity compared with the control group. Although the current study did not conduct inferential analysis on this matter, the results somewhat supported those of the previous studies that older adults with higher levels of education and socioeconomic status tended to have higher physical activity levels (Eronen et al., 2016; Zainol et al., 2016). For instance, education levels were associated with an average increase of 50% in hours of accelerometer use among older adults compared to counterparts with lower education levels (Zainol et al., 2016). The results of the present and previous studies consistently show that the sociodemographic factors influence physical activity levels in older adults, which also affect physical and health conditions.

The results of the blood lipids profile showed significant improvements after the exercise intervention in total cholesterol, triglycerides, and VLDL, which is consistent with the previous studies. For instance, Martins et al. (2010) found that physical activity participation contributed to older adults’ improvements in levels of triglycerides, total cholesterol, HDL, and LDL. In another study, Ho et al. (2012) found that obese older adults who participated in a 12-week aerobic exercise program improved in HDL, whereas counterparts who did not showed no changes. A result found in the present study and which was not reported in the results of the mentioned studies was the significant effect on VLDL. This finding is important because it reinforces the usefulness of physical exercise to increase the catabolism of VLDLs that help to transport mainly triglycerides to the tissues, that is, at higher levels greater risk of accumulation of fat in the arteries limiting the flow of blood rich in oxygen to the body and as a consequence an increase in the risk of heart disease (Carvajal, 2014).

The present study revealed that the 12-week exercise participation resulted in improvements in systolic and diastolic blood pressure. However, the results do not necessarily support the previous studies. For instance, Martins et al. (2010) found that a 16-week multicomponent exercise program (e.g., using aerobic and strength exercise) affected decreases in older adults’ diastolic but not systolic blood pressure. Also, (Finucane et al., 2010) found that a 12-week aerobic exercise program with 36 sessions utilizing cycle ergometers was not effective for both systolic and diastolic blood pressure. The results may be because the type of exercise used was low impact and involved fewer muscle groups, whereas in the present study, most exercises used, such as walking, jogging, and dancing, involved larger muscle groups.

The exercise program had a positive effect on the nutritional status of the AM however there were no significant differences between the initial evaluation and final evaluation in the EG, this may be due to the fact that among the parameters contemplated in the state of nutrition, those referring to anthropometric measurements also did not show significant improvements. In terms of anthropometric variables, the older adults in this study reduced body weight and BMI through exercise participation but not to a statistically significant degree. These results are consistent with previous studies (Martins et al., 2010; Villareal et al., 2011). For instance, Villarreal et al. (2011) provided 12-month interventions to older adults with obesity while assigning them to one of four groups: a diet, an exercise, a diet-exercise, or a control group. The results revealed that the participants in the diet and the diet-exercise groups significantly decreased in body weight; however, those who were in the exercise and the control groups did not. Martins et al. (2010) found that 16-week interventions using either aerobic or strength training were not effective for improvements in older participants’ BMI and body weight. Based on the results of the present and previous studies, it seems that interventions using both exercise and nutrition programs are more effective than an exercise-only intervention for older adults’ weight management. However, research may need to be continued to find if an exercise-only intervention is effective for older adults’ weight management and they will have to be strengthened with periodic dietary controls because physical activity alone is not enough to reduce fat levels and increase muscle mass in older adults.

The current study revealed that 12-week exercise participation was effective for improvements in depressive symptoms and self-esteem. These results are consistent with the previous studies, which demonstrated that exercise participation contributed to the reduction of depressive symptoms and anxiety (Carek et al., 2011; Dinas et al., 2011). Also, with the results of Salguero et al. (2011) who studied the relationship between physical activity and health-related quality of life and depressive symptoms in older community residents and institutionalized older adults in northern Spain. The results of the level of physical activity were related to different domains of the physical and mental components of health-related quality of life and the decrease in depressive symptoms. In the present study, only older adults with disease improved self-esteem and spent longer on physical activity, which is consistent with the findings from the previous studies that older adults continued to be physically active when they had better self-esteem (Bergland et al., 2010; García and Troyano, 2013).

The older adults of the present study who participated in the exercise program increased their physical activity levels. The group participating in the physical activity intervention spent 40 more minutes per week on physical activity than the other group. The results are consistent with the previous study (Pahor et al., 2014) that older adults who were provided with a physical activity intervention increase physical activity levels compared with those who were provided with health education. Mama et al. (2015) also carried out a systematic review of physical activity interventions with an emphasis placed on psychosocial factors provided for African Americans and Hispanics older adults. The results showed that the interventions that included psychosocial factors such as using strategies to increase motivation or self-efficacy tended to contribute to participants’ increase in physical activity and achievement of a higher attendance record in the physical activity.

One of the limitations of the study was the small sample size, it is necessary to expand it and have a homogeneous distribution by sex, also by the duration of the program: interventions of longer duration that allow observing changes in other anthropometric variables and physiological. In addition, the calculation of the magnitude of the effect would be more accurate with larger populations. Another limitation was that in this study the use of music was limited only as a structural resource in the physical activity session and as an accompaniment during the development of the exercises, so the effect on the study variables was not measured. However, research in neuropsychology has used music for other purposes such as controlling emotions, improving quality of life, self-care, and behavior, through the reactions to specific musical signals such as melody, harmony, tempo, doorbell music, among others (Costa et al., 2012; Castillo et al., 2014; Fernández-Sotos et al., 2016). Therefore, it is recommended for future research to consider determining the influence of music on physical and psychological variables.

## Conclusions

The current study supports that older adults who participate in multidimensional exercise programs (e.g., theory-based integrative biopsychosocial factors) improve their cardiovascular function, their self-esteem and depress the depressive symptoms. The increase in physical activity levels can contribute to older adults’ improvement in physical and mental health and making health-related behavioral change. Therefore, it is recommended that integrating exercise and physical activity into daily life can be a proactive, economical approach for older adults with various health conditions to maintain and improve their physical and psychological health and well-being. Moreover, it evaluates the adherence of the elderly to the practice of physical activity to strengthen the psychosocial factors that influence health behaviors and monitor the participants after finishing their participation in the program to verify the specific moment in which the stopping training produces significant changes in the factors of healthy physical condition.

## Data Availability Statement

All datasets generated for this study are included in the article/Supplementary Material.

## Ethics Statement

The studies involving human participants were reviewed and approved by the research that was authorized by the Comité Institucional de Bioética of the Instituto Técnico de Sonora and complied with the Official Mexican Standard NOM-012-SSA3-2012, which establishes the criteria for performing human research projects in health. The patients/participants provided their written informed consent to participate in this study.

## Author Contributions

NB-C is responsible for the study design and supervision of the exercise program application. AC-Z collaborated on the survey and analysis of the data. RC-C collaborated in the design of the study, supervised the data collection and the writing of the manuscript. NB-S and MD-O collaborated in the study design and the manuscript review.

### Conflict of Interest

The authors declare that the research was conducted in the absence of any commercial or financial relationships that could be construed as a potential conflict of interest.

## Abbreviations

HPM: Health promotion model
BMI: Body mass index
SBP: Systolic blood pressure
DBP: Diastolic blood pressure
GDS-15: Geriatric depression scale 15 items
RSES: Rosenberg self-esteem scale
SEL: Socioeconomic level
AMAI: Mexican Association of Marketing and Opinion Intelligence Agencies
CAFAM: Physical activity questionnaire for the elderly adult
METs: Metabolic equivalents.
