# Supplementary material for: The Effects of a Multidimensional Exercise Program on Health Behavior and Biopsychological Factors in Mexican Older Adults
Source: Front Psychol. 2020 Jan 24;10:2668. doi: 10.3389/fpsyg.2019.02668 (PMC6993582; doi:10.3389/fpsyg.2019.02668)
Supplement: Supplementary file 1 [file Data_Sheet_1.docx]

**Supplementary material**

**The effects of a multidimensional exercise program on health behavior and biopsychosocial factors in Mexican older adults**

Norma Angélica Borbón-Castro^1^, Andrés Aquilino Castro-Zamora^2^, Rosa María Cruz-Castruita^3^ *, Nancy Cristina Banda-Sauceda^4^, Manuel Francisco De La Cruz-Ortega^1^.

^1^ Programa educativo Licenciado en Entrenamiento Deportivo. Universidad Estatal de Sonora, Unidad Académica Hermosillo.

^2^ Programa educativo Licenciado en Nutrición Humana, Universidad Estatal de Sonora. Hermosillo, Sonora, México.

^3^ Universidad Autónoma de Nuevo León, Facultad de Organización Deportiva. San Nicolás de los Garza, Nuevo León, México.

^4^, Universidad Autónoma de Nuevo León, Facultad de Nutrición y Salud Pública. San Nicolás de los Garza, Nuevo León, México.

*** Correspondence:**

Corresponding author

[rosa.cruzcst@uanl.edu.mx](mailto:rosa.cruzcst@uanl.edu.mx)

Table 1

*Modules and goals of the exercise program*

| Modules | Goals |
| --- | --- |
| A | Develop aerobic endurance through light walk exercises and rhythmic activities. In addition, muscular endurance through strengthening the upper and lower body. |
| B | Develop aerobic endurance through light walk exercises, rhythmic activities and group dynamics. As well as increase joint amplitude and strengthen the upper and lower train through exercises performed using the circuit method. |
| C | Improve aerobic endurance through rhythmic activities, group dynamics and fartlek walks. In addition to strengthening the upper and lower train through exercises with self-loading and resistance bands. |
| D | Increase aerobic endurance through fartlek-type walks, rhythmic activities and group dynamics. Also improve flexibility, balance, coordination and agility. As well as strengthening the upper and lower train through exercises with self-loading and resistance bands. |
| E | Increase aerobic endurance through walking tests, fartlek walks, rhythmic activities and group dynamics. Also improve balance, coordination and agility. As well as increase the muscular resistance of the upper and lower train through exercises with self-loading and resistance bands. |
| F | Improve agility through reaction speed exercises, improve balance through monopodial exercises and increase upper end muscle resistance through resistance band exercises, as well as aerobic endurance through speed walking and rhythmic exercises. |

Table 2

*Descriptions of the biological and social factors in older adults*

| Variable | EG (*n* = 23) | | CG (*n* = 22) | |
| --- | --- | --- | --- | --- |
|  | *f* | % | *f* | % |
| Biological | | | | |
| Health status |  |  |  |  |
| Healthy | 4 | 17.39 | 10 | 45.45 |
| One disease | 8 | 34.78 | 4 | 18.18 |
| two or more disease | 11 | 47.82 | 8 | 36.36 |
| Medication use |  |  |  |  |
| Does not use drugs | 5 | 21.73 | 8 | 36.36 |
| One drug | 5 | 21.73 | 6 | 27.27 |
| Two or more drugs | 13 | 56.52 | 8 | 36.36 |
| Sociocultural | | | | |
| Affiliated to a health service |  |  |  |  |
| Yes | 23 | 100 | 22 | 100 |
| No | 0 | 0 | 0 | 0 |
| Type of health service |  |  |  |  |
| Government | 23 | 100 | 20 | 90.87 |
| Private | 0 | 0 | 2 | 9.09 |
| Marital status |  |  |  |  |
| Single | 3 | 13.04 | 4 | 18.18 |
| Married |  |  |  |  |
| Widowed | 5 | 21.73 | 5 | 22.72 |
| Educational level |  |  |  |  |
| Elementary school | 7 | 30.43 | 12 | 54.54 |
| High school | 6 | 26.08 | 4 | 18.18 |
| College | 10 | 43.47 | 6 | 27.27 |
| Socioeconomic level |  |  |  |  |
| Low | 2 | 8.69 | 3 | 13.63 |
| Middle | 9 | 39.13 | 14 | 63.63 |
| High | 12 | 52.17 | 5 | 22.72 |

*Note:* EG = Experimental Group; CG = Control Group; *f* = frequency; % = percentage; *n* = sample.

Table 3

*Comparison between test of biological factor in older adults*

| Factor | G | Pretest  (M + SD) | *p* | Posttest  (M + SD) | *p* | Pretest vs Posttest | *d* | r | R^2^ |
| --- | --- | --- | --- | --- | --- | --- | --- | --- | --- |
| Glucose (mg/dl) | EG | 81.75 ± 10.79 | 0.61 | 87.45 ± 13.75 | 0.75 | 0.14 | -0.46 | -0.22 | 0.048 |
|  | CG | 84.10 ± 17.31 |  | 85.94 ± 17.02 |  | 0.74 | -0.10 | -0.05 | 0.002 |
| HbA1 (%) | EG | 6.74 ± 0.62 | 0.41 | 6.66 ± 0.51 | 0.63 | 0.66 | 0.14 | 0.07 | 0.004 |
|  | CG | 6.88 ± 0.48 |  | 6.73 ± 0.35 |  | 0.26 | 0.35 | 0.17 | 0.028 |
| TC (mg/dl) | EG | 181.69 ± 34.30 | 0.23 | 160.95 ± 30.53 | 0.56 | 0.03^*^ | 0.63 | 0.30 | 0.09 |
|  | CG | 169.63 ± 32.42 |  | 166.36 ± 32.02 |  | 0.73 | 0.10 | 0.05 | 0.002 |
| TG | EG | 128.95 ± 38.07 | 0.08 | 103.77 ± 33.18 | 0.33 | 0.02^*^ | 0.70 | 0.33 | 0.108 |
|  | CG | 104.94 ± 48.11 |  | 117.22 ± 55.36 |  | 0.45 | -0.23 | -0.11 | 0.012 |
| HDL (mg/dl) | EG | 44.04 ± 3.12 | 0.47 | 45.24 ± 2.91 | 0.60 | 0.18 | -0.39 | -0.19 | 0.036 |
|  | CG | 44.76 ± 3.58 |  | 45.68 ± 2.61 |  | 0.33 | -0.29 | -0.14 | 0.019 |
| LDL (mg/dl) | EG | 109.64 ± 32.80 | 0.23 | 93.65 ± 32.49 | 0.69 | 0.10 | 0.48 | 0.23 | 0.052 |
|  | CG | 98.30 ± 29.83 |  | 97.2 ± 27.98 |  | 0.90 | 0.03 | 0.01 | 0.000 |
| VLDL | EG | 26.60 ± 8.22 | 0.18 | 20.75 ± 6.63 | 0.33 | 0.01^**^ | 0.78 | 0.36 | 0.129 |
|  | CG | 22.49 ± 11.52 |  | 23.44 ± 11.07 |  | 0.78 | -0.08 | -0.04 | 0.001 |
| Body weight (kg) | EG | 72.07 ± 11.69 | .398 | 71.53 ± 11.20 | .369 | .872 | 0.04 | 0.02 | 0.000 |
|  | CG | 69.16 ± 10.88 |  | 68.54 ± 10.57 |  | .853 | 0.05 | 0.02 | 0.000 |
| BMI, kg/m^2^ | EG | 29.14 ± 5.11 | .159 | 28.96 ± 5.07 | .137 | .903 | 0.03 | 0.01 | 0.000 |
|  | CG | 27.13 ± 3.92 |  | 26.87 ± 3.75 |  | .834 | 0.06 | 0.03 | 0.000 |
| SBP (mm/Hg)^†^ | EG | 139.13 ± 16.49 | .102 | 118.04 ± 5.78 | .069 | .001* |  |  |  |
|  | CG | 129.09 ± 19.49 |  | 124.31 ± 21.67 |  | .310 |  |  |  |
| DBP (mm/Hg)† | EG | 80.00 ± 0 | .202 | 76.84 ± 4.77 | .694 | .027* | 0.93 | 0.42 | 0.176 |
|  | CG | 76.36 ± 8.4 |  | 75.68 ± 9.7 |  | .637 | 0.07 | 0.03 | 0.000 |
| Nutritional status | EG | 25.54 ± 2.46 | 0.15 | 26.56 ± 2.03 | 0.01^**^ | 0.13 | -0.45 | -0.22 | 0.048 |
|  | CG | 24.27 ± 3.42 |  | 24.43 ± 3.55 |  | 0.88 | -0.04 | -0.02 | 0.000 |

*Note:* M = Medium; SD = standard deviation; *p* = Value of significance; *d* = Cohen´s d; R^2^ = Determination coefficient; G = Group; GE = Experimental group; GC = Control group; HbA1c = glycosylated hemoglobin; TC = Total cholesterol; TG = Triglycerides; HDL = High density lipoproteins; LDL = Low density lipoproteins; VLDL = Very low density lipoproteins; BMI = body mass index; SBP = Systolic blood pressure; DBP = Diastolic blood pressure

* p <0.05; ** p <0.01; *** p <0.001

^†^ Data with non-normal distribution.

Table 4

*Comparison between test of psychological factor and health behavior in older adults*

| Factor |  | Pretest  Mean ± SD | *p* | Posttest  Mean ± SD | *p* | Pretest vs Posttest | d | r | R^2^ |
| --- | --- | --- | --- | --- | --- | --- | --- | --- | --- |
| Psychological | | | | | | | | | |
| Depression^†^ | EG | 1.91 ± 2.60 | .010* | 0.60 ± 1.03 | .001* | .002* |  |  |  |
|  | CG | 3.72 ± 2.91 |  | 4.09 ± 2.70 |  | .238 |  |  |  |
| Self-esteem | EG | 23.42 ± 1.50 | .855 | 24.91 ± 1.83 | .005* | .005* | -0.89 | -0.40 | 0.16 |
|  | CG | 23.54 ± 2.52 |  | 23.40 ± 1.53 |  | .824 | 0.06 | 0.03 | 0.00 |
| Health behavior | | | | | | | | | |
| PA level (METs) | EG | 3.22 ± 0.77 | .169 | 7.30 ± 1.12 | .001* | .001* | -4.24 | -0.90 | 0.81 |
|  | CG | 3.71 ± 1.48 |  | 3.51 ± 1.23 |  | .634 | 0.14 | 0.07 | 0.00 |

*Note:* SD = standard deviation; *p* = significance value; *d* = Cohen´s d; R^2^ = Determination coefficient; GE = Experimental group; GC = Control group; PA = physical activity; METs = metabolic equivalents.

* *p* < 0.05; ** *p* < 0.01; ****p* < 0.001

^†^ Data with non-normal distribution.

Table 5

*Comparison of the biological and psychological factors and health behavior between evaluation and by healthy and disease older adults*

| Factor | Pretest  Mean ± SD | *p* | Posttest  Mean ± SD | *p* | *p*  Pretest vs Posttes |
| --- | --- | --- | --- | --- | --- |
| Biological | | | | | |
| Body weight (kg) | |  |  |  |  |
| Healthy | 65.56 ± 8.46 | .038* | 65.09 ± 8.43 | .035* | .883 |
| With disease | 73.07 ± 11.74 |  | 72.45 ± 11.22 |  | .833 |
| BMI, kg/m^2^ |  |  |  |  |  |
| Healthy | 25.24 ± 2.54 | .002* | 25.06 ± 2.52 | .002* | .853 |
| With disease | 29.64 ± 4.80 |  | 29.40 ± 4.70 |  | .851 |
| SBP (mm/Hg)† |  |  |  |  |  |
| Healthy | 129.28 ± 23.35 | .220 | 112.50 ± 19.68 | .033* | .043* |
| With disease | 136.45 ± 15.82 |  | 125.00 ± 12.24 |  | .013* |
| DBP (mm/Hg)† |  |  |  |  |  |
| Healthy | 76.42 ± 8.41 | .436 | 72.69 ± 9.70 | .110 | .449 |
| With disease | 78.96 ± 4.88 |  | 77.85 ± 6.29 |  | .546 |
| Psychological | | | | | |
| Depression† |  |  |  |  |  |
| Healthy | 2.21 ± 2.04 | .6233 | 2.50 ± 2.34 | .432 | .751 |
| With disease | 3.06 ± 3.18 |  | 2.22 ± 2.83 |  | .168 |
| Self-esteem |  |  |  |  |  |
| Healthy | 24.21 ± 2.29 | .109 | 23.53 ± 1.89 | .117 | .414 |
| With disease | 23.13 ± 1.88 |  | 24.50 ± 1.77 |  | .005* |
| Health behavior | | | | | |
| PA level (METs) | |  |  |  |  |
| Healthy | 3.87 ± 1.66 | .114 | 4.66 ± 2.02 | .116 | .269 |
| With disease | 3.27 ± 0.86 |  | 5.80 ± 2.27 |  | .001* |

*Note:* SD = standard deviation; BMI = body mass index; PA = physical activity; SBP = systolic blood pressure; DBP = diastolic blood pressure; METs = metabolic equivalents; Kg = Kilograms; squared meters; mm/Hg = millimeters of mercury

*p < 0.05

^†^ Data with non-normal distribution.
